# Changes in retinal circulation and choroidal thickness in patients with acute myeloid leukemia detected by optical coherence tomography angiography

**DOI:** 10.3389/fmed.2023.1117204

**Published:** 2023-03-13

**Authors:** Ling Yang, Yanwei Chen, Yunxiang Zhang, Ting Shen, Xi Shen

**Affiliations:** ^1^Department of Ophthalmology, RuiJin Hospital Lu Wan Branch, Shanghai Jiaotong University School of Medicine, Shanghai, China; ^2^Department of Ophthalmology, RuiJin Hospital, Shanghai Jiaotong University School of Medicine, Shanghai, China; ^3^Department of Hematology, RuiJin Hospital, Shanghai Jiaotong University School of Medicine, Shanghai, China

**Keywords:** acute myeloid leukemia, leukemic retinopathy, retinal circulation, choroidal thickness, optical coherence tomography angiography

## Abstract

**Purpose:**

To investigate changes in retinal circulation and the choroid in patients with acute myeloid leukemia (AML) in the acute and remission stages, to analyze the correlation between retinal circulation and laboratory parameters, and to assess risk factors associated with leukemic retinopathy.

**Methods:**

Forty-eight patients (93 eyes) with AML were enrolled and divided into two groups according to fundus examination findings: the retinopathy and no retinopathy groups. Patients underwent eye measurements before treatment and after remission. Macular vessel density (VD), perfusion density (PD), foveal avascular zone (FAZ), and choroidal thickness (ChT) were measured using optical coherence tomography angiography. Patients with healthy eyes were recruited as control participants.

**Results:**

Patients with leukemic retinopathy had higher measurements of white blood cells (WBCs), circulating blasts, fibrin degradation products, and cross-linked fibrin degradation products (D-dimer) and a lower hemoglobin (HB) count (*p* < 0.05). In the acute phase of the disease, the VD and PD were lower and the ChT was thicker in patients with AML than in controls (*p* < 0.05), irrespective of the presence of leukemic retinopathy; however, the patients were partially recovered in the remission stage. The VD was lower in patients with higher WBC (*r* = −0.217, *p* = 0.036), D-dimer (*r* = −0.279, *p* = 0.001), fasting blood glucose (FBG) (*r* = −0.298, *p* = 0.004) and triglyceride (*r* = −0.336, *p* = 0.001) levels. The FAZ area was negatively correlated with HB (*r* = −0.258, *p* = 0.012).

**Conclusion:**

Patients with AML appear to have subclinical retinal perfusion loss and choroidal thickening in the acute phase of the disease, but this is reversible. Injury to bone marrow function may cause a decrease in retinal perfusion. Leukemic retinopathy is associated with abnormal hematologic parameters and coagulopathy.

## Introduction

Leukemia is a malignant tumor of the bone marrow that causes an abnormal production of white blood cells (WBCs), affecting multiple organs (1). Ocular complications are common in patients with leukemia, especially acute myeloid leukemia (AML) (2). The retina is the most frequently affected site. Direct infiltration of tumor cells or hematologic abnormalities (thrombocytopaenia, anemia, and high viscosity) can lead to leukemic retinopathy (3–5). Retinal hemorrhage is the most common sign (2, 6, 7), and retinal vein dilation, cotton lint spots, vitreous hemorrhage, and papilledema can also manifest. Previous *ex vivo* studies have demonstrated retinal microvascular involvement (8). The choroid is also constantly affected by leukemic cell infiltration and can present as a serous retinal detachment (9). Ocular involvement may be the initial manifestation or first sign of systemic disease recurrence (10). To obtain more subclinical evidence of ocular involvement and early detection of disease progression, we used the quantitative analysis function of optical coherence tomography (OCTA) to investigate the changes in retinal circulation and the choroid in patients with AML in the acute and remission stages, correlated retinal circulation with laboratory parameters, and assessed some risk factors associated with leukemic retinopathy.

## Method

### Study design and participants

This was a prospective study. All patients with AML were recruited from the Department of Hematology at Ruijin Hospital, Shanghai Jiao Tong University School of Medicine from January 2021 to October 2021. Written informed consent was obtained from each participant. The design and procedure of this research adhered to the principles of the Declaration of Helsinki. The Institutional Review Board of Ruijin Hospital Luwan Branch authorized this study.

All patients with AML were aged >18 years, newly diagnosed, and to undergo systemic chemotherapy with idarubicin combined with cytarabine (IA regimen). The exclusion criteria were as follows: (1) opacity of the refractive stroma or macular lesions affecting fundus imaging; (2) fundus vascular diseases such as glaucoma, uveitis, high myopia, diabetic retinopathy, and age-related macular degeneration; (3) history of ocular surgical procedures; and (4) other severe systemic diseases. Age-matched healthy volunteers seeking physical examinations were enrolled as the control group during the same time period. According to fundus examination findings, patients with AML were divided into two groups: the retinopathy and no retinopathy groups. All patients were followed up until they completed chemotherapy. After hematologists’ evaluation, patients who achieved complete remission (CR) were selected for further research.

### Measurement of clinical examination

All participants underwent a complete ophthalmic examination, including best-corrected visual acuity, computer optometry, intraocular pressure (IOP) measurement with Goldmann applanation tonometry, axial length (AL), slit lamp examination, fundus examination in artificial mydriasis, fundus photography, and OCTA scans of the macular area. Fundus photography and OCTA scans were repeated after CR.

Age, sex, and medical and ocular history were recorded. Laboratory parameters from peripheral blood samples, including WBCs (×109/L), circulating blasts (blast) (%), hemoglobin (HB) (g/dL), platelets (PLTs) (g/L), fibrinogen (Fg) (g/L), fibrin degradation products (FDP) (mg/L), cross-linked fibrin degradation products (D-dimer) (mg/L), fasting plasma glucose (FPG) (mmol/L), triglyceride (TG) (mmol/L), and total cholesterol (TC) (mmol/L) levels were recorded.

Macular OCTA scans were captured using Cirrus HD Oct 5,000 software version 9.5.2. (Carl Zeiss Meditec) and analyzed using software version 10.0. The superficial macular vessel density (VD) was obtained by angiography (6 × 6 mm), and choroidal thickness (ChT) was obtained using the HD Cross mode. To reduce the influence of motion artifacts, a tracking technique was used. Parameters, including the foveal avascular zone (FAZ), VD, and perfusion density (PD), were calculated to assess the superficial retinal vessels (from the inner limiting membrane layer to the inner plexiform layer) using the manufacturer’s vascular measurement software. VD is the linear length of the vessel divided by the selected area, and PD is the area of vessel distribution divided by the selected area. ChT is the distance from the high reflection line of the Bruch membrane to the line of the inner surface of the sclera. ChT was measured manually in both the horizontal and vertical directions including points of the fovea, 1.0 mm from the fovea in the superior, inferior, nasal, and temporal directions. Each point was measured three times and averaged. One skilled doctor who was masked to the patient’s systemic and ocular data performed the measurements.

**Figure 1:**
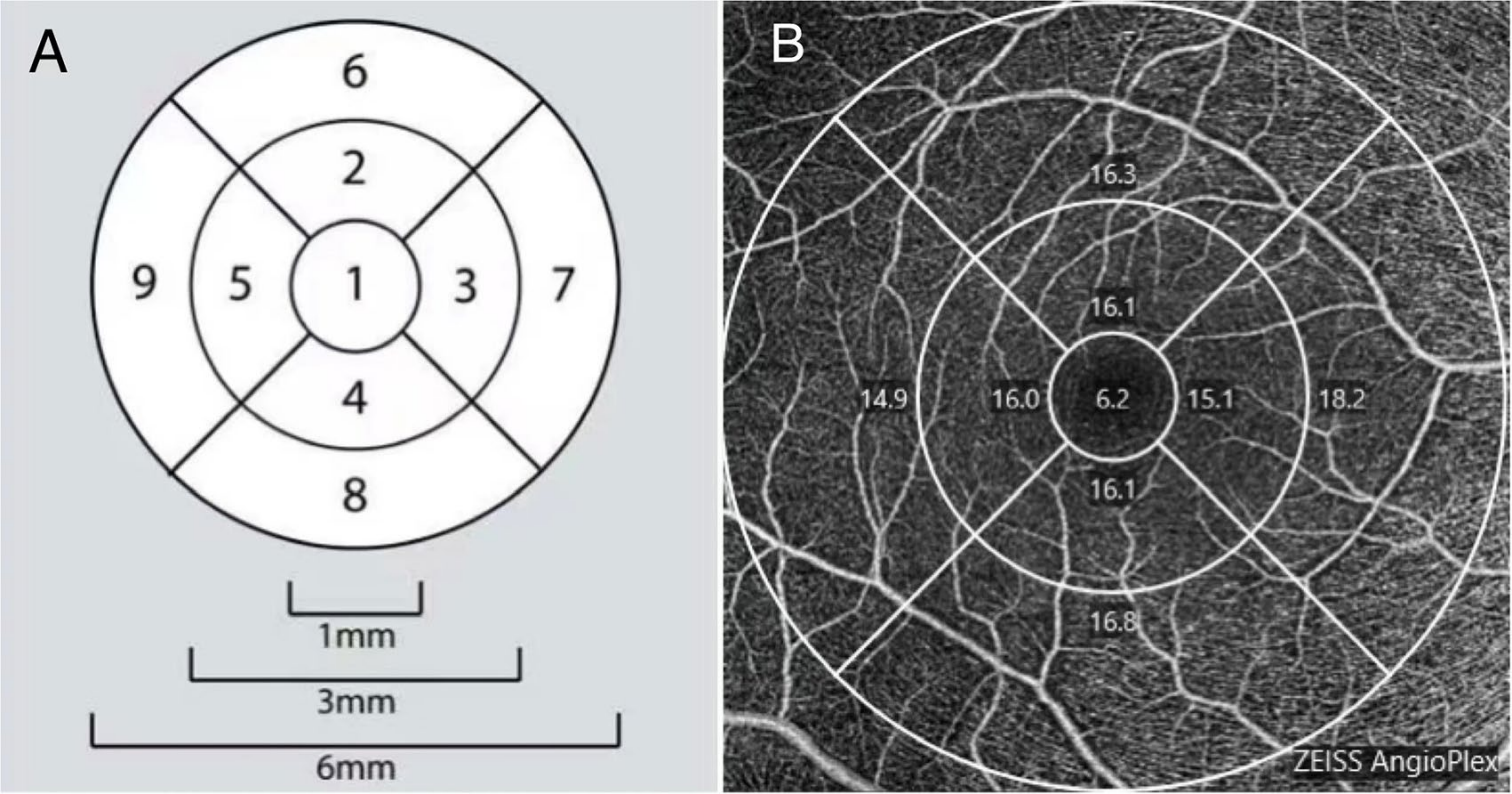
**(A)** A demonstration diagram of the partition in macular area. **(B)** Example images of macular vessel density measurement based on optical coherence tomography angiography (OCTA).

The VD map of the macula was a 6-mm-diameter circular area divided into nine sections with three concentric rings according to the Early Treatment Diabetic Retinopathy Study map. The inner ring was 1.0 mm in diameter, middle ring was 3 mm, and outer ring was 6 mm. VD and PD were automatically calculated. The central subfield was defined as a disk-shaped region of 1-mm diameter centered on the fovea (region 1). The value of the inner subfield was the average of each inner quadrant of an annulus centered on the fovea with an inner diameter of 1 mm and an outer diameter of 3 mm (regions 2, 3, 4, and 5). The value of the outer subfield was the average of each outer quadrant of an annulus centered on the fovea with an inner diameter of 3 mm and an outer diameter of 6 mm diameter (regions 6, 7, 8, and 9; Figure 1). One skilled doctor obtained all OCT scans. Images with signal intensities higher than six were selected for the analysis.

### Statistical analysis

Statistical analysis was performed using SPSS 20.0 (IBM Corporation, Chicago, IL, USA). All data were tested for normality using the Kolmogorov–Smirnov test. Normally distributed data are presented as means ± standard deviations (SD), and partial non-normally distributed data are presented as medians with interquartile ranges. One-way analysis of variance was used to compare data with a normal distribution and equal variance among the three groups. The least SD test was subsequently performed for group comparisons. Otherwise, a Kruskal–Wallis test was adopted, and a Mann–Whitney *U* test was used for group comparisons. The paired *t*-test or Wilcoxon signed-rank test was used to assess the differences in data before and after chemotherapy. Spearman’s correlation coefficients were calculated to evaluate the relationships between different clinical parameters. A chi-square test was used to assess the female to male ratio differences among the three groups. Results were considered statistically significant at *p* < 0.05.

## Result

A total of 71 participants were enrolled, including 23 healthy individuals (46 eyes) and 48 patients with AML (93 eyes). Forty eyes belonging to 21 patients with AML were diagnosed with leukemic retinopathy. Fundus examination showed varying degrees of fundus bleeding and tortuous venous dilatation in all these eyes, some accompanied by cotton wool spots, hard exudation, and optic disc edema. None of the macular regions were involved. Only one patient complained of vision loss, and only six had symptoms of shadows or flying mosquitoes. The other 27 patients (53 eyes) had no leukemic retinopathy. The best-corrected visual acuity was 1.0 in 86 eyes (92.5%), 0.9 in two eyes (0.02%), and 0.8 in five eyes (0.05%).

The basic characteristics of all participants are shown in Table 1. There were no statistically significant differences in age, sex distribution, IOP, and AL among the three groups (*p* = 0.714, *p* = 0.994, *p* = 0.306, and *p* = 0.155, respectively).

**Table 1:** Characteristics of the study population.

	Retinopathy	No retinopathy	Normal	*P*-value
Age (years)	43.2 ± 13.6	45.0 ± 13.2	46.2 ± 10.0	0.714
Sex (F/M)	9/12	12/15	10/13	0.994
IOP (mmHg)	16.2 ± 2.2	16.6 ± 2.0	16.7 ± 1.9	0.306
AL (mm)	23.50 ± 1.22	23.72 ± 0.94	23.19 ± 1.02	0.155

Compared with patients without leukemic retinopathy, patients with leukemic retinopathy had a higher count of WBCs (*p* = 0.03) and circulating blasts (*p* = 0.023), and a lower count of HB (*p* < 0.001). Moreover, the FDP and D-dimer levels in the retinopathy group were also higher (*p* = 0.038 and *p* = 0.046, respectively). No significant differences were observed in bleeding symptoms, and PLT, FPG, TG and TC levels (Table 2).

**Table 2:** Comparison of basic information and hematologic parameters between the retinopathy and no retinopathy group.

	Retinopathy	No retinopathy	*P*-value
Other bleeding Symptoms(with/without)	10/11	8/19	0.164
WBC	13.67(4.3,24.8)	3.9(2,10.5)	0.03^*^
HB	74 ± 23	100 ± 23	<0.001^*^
PLT	48(23,80)	75(27,147)	0.122
Blast(%)	46 ± 27	29 ± 23	0.023^*^
Fg	3.1 ± 0.9	3.2 ± 1.0	0.895
FDP	6.1(2.8,10.1)	3.0(2.5,5.9)	0.038^*^
D-dimer	1.7(0.51,3.13)	0.6(0.36,1.53)	0.046^*^
FBG	5.19 ± 0.62	5.38 ± 1.34	0.567
TG	1.45 ± 0.62	1.46 ± 0.66	0.951
TC	3.51 ± 0.90	3.86 ± 1.13	0.255

The assessment of superficial retinal vessels measured using OCTA is summarized in Table 3. Patients with AML had lower VD and PD measurements than did healthy individuals (all *p* < 0.05), irrespective of the presence of leukemic retinopathy, but no significant difference in the central subfield was observed. ChT was thicker in patients with AML (*p* < 0.05), regardless of the presence or absence of leukemic retinopathy, except in the superior. The FAZ areas among the three groups were not significantly different.

**Table 3:** Optical coherence tomography angiography (OCTA) analysis results in different study groups.

	Retinopathy	No retinopathy	Normal	*P*-value	P1	P2	P3
Vascular density (mm^−1^)							
Central	6.5 ± 2.9	5.6 ± 2.5	6.9 ± 4.6	0.425			
Inner	14.8 ± 2.5	15.3 ± 2.9	16.9 ± 1.9	<0.001^*^	0.173	<0.001^*^	0.003^*^
Outer	16 ± 2.1	16.4 ± 2.1	17.6 ± 1.6	<0.001^*^	0.159	<0.001^*^	0.001^*^
Perfusion density							
Central	0.160 ± 0.101	0.144 ± 0.087	0.166 ± 0.107	0.575			
Inner	0.340 ± 0.071	0.362 ± 0.077	0.390 ± 0.068	0.001^*^	0.073	<0.001^*^	0.034^*^
Outer	0.396 ± 0.062	0.399 ± 0.055	0.441 ± 0.039	<0.001^*^	0.759	<0.001^*^	<0.001^*^
FAZ area (mm^2^)	0.24 ± 0.11	0.25 ± 0.13	0.26 ± 0.13	0.687			
Choroidal thickness (um)							
Subfoveal	266 ± 49	257 ± 45	238 ± 36	0.012^*^	0.341	0.004^*^	0.038^*^
Superior	249 ± 48	240 ± 48	224 ± 26	<0.024^*^	0.330	0.008^*^	0.066
Temporal	250 ± 45	242 ± 42	223 ± 27	<0.005^*^	0.369	0.002^*^	0.016^*^
Inferior	245 ± 49	244 ± 46	226 ± 26	<0.062	0.914	0.042^*^	0.041^*^
Nasal	237 ± 47	232 ± 43	212 ± 23	<0.01^*^	0.592	0.016^*^	0.02^*^

P1: *P*-value for the comparison group between retinopathy group and no retinopathy group.

P2: *P*-value for the comparison group between retinopathy group and normal controls.

P3: *P*-value for the comparison group between no retinopathy group and normal controls.

^*^Statistically meaningful.

**Figure 2:**
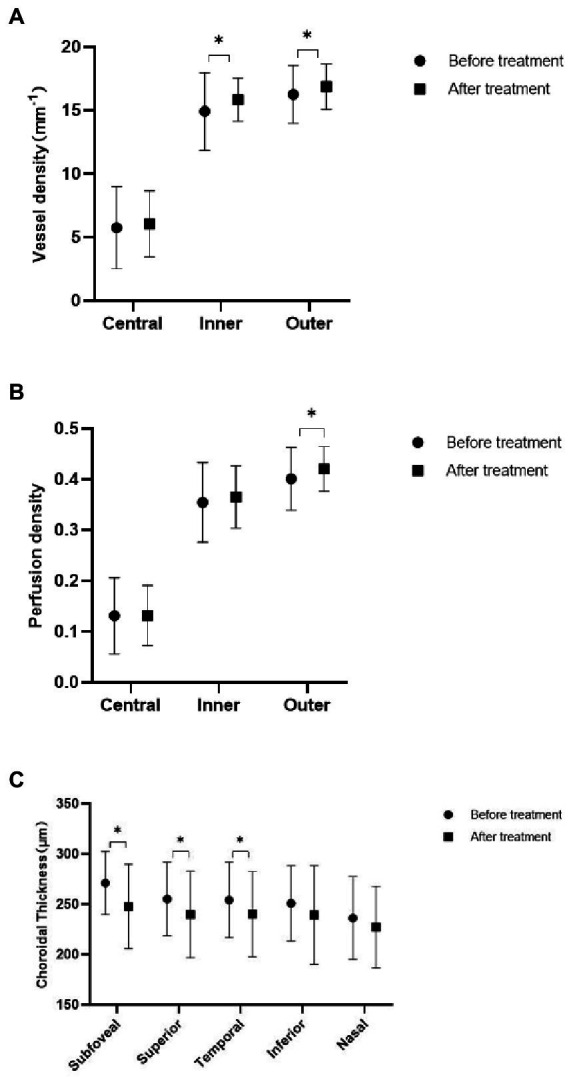
Comparison of acute myeloid leukemia (AML) patients before and after treatment. **(A)** Three zones of macular vessel density before and after treatment. **(B)** Three zones of macular perfusion density before and after treatment. **(C)** Choroidal thickness at five measurement points before and after treatment.

**Figure 3:**
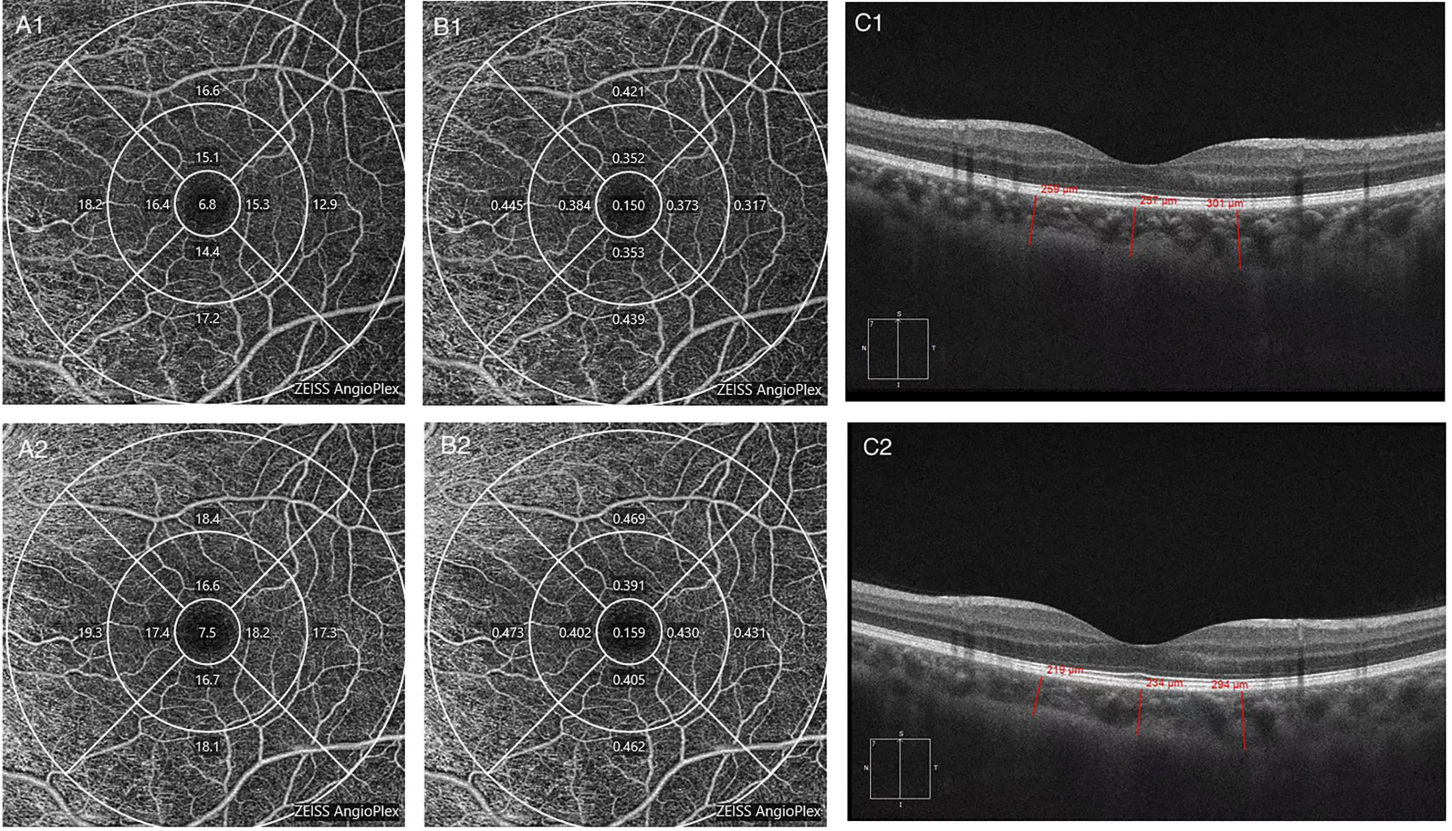
The changes in a patient imaged with optical coherence tomography angiography (OCTA) in the active phase of acute myeloid leukemia (AML) and after remission. **(A1)** Macular vessel density before treatment. **(A2)** Macular vessel density after treatment. **(B1)** Macular perfusion density before treatment. **(B2)** Macular perfusion density after treatment. **(C1)** Choroidal thickness before treatment. **(C2)** Choroidal thickness after treatment.

After chemotherapy, the patients underwent OCTA and fundus photography again before discharge. The mean follow-up period was 46 days. Two patients died, and 15 were lost to follow-up during this period. Eight patients did not achieve CR before being discharged according to the hematologists’ assessment. Finally, 23 patients (44 eyes) were enrolled for longitudinal comparison. Among them, 18 eyes were from the retinopathy group, and the rest were from the no retinopathy group. After remission, fundus hemorrhage decreased in two eyes, increased in one eye, and showed no significant change in 41 eyes. VD in the inner and outer subfields increased (*p* = 0.043 and *p* = 0.036, respectively), but no substantial change in the central subfield was observed. PD increased only in the outer subfield (*p* = 0.04), and the ChT decreased at most of the measurement points, except for the nasal and inferior subfields (*p* = 0.001, *p* = 0.016, *p* = 0.023; Figure 2). Figure 3 shows a clinical example of the macular perfusion and choroid changes in a patient imaged with OCTA in the active phase of AML and after remission. This patient was a 20-year-old boy. The first inspection time was July 21 and the review time was August 19. The visual acuity of his left eye was 1.2 in both exams. No leukemic retinopathy was observed before and after treatment. A1, A2, B1 and B2 show that macular perfusion normalized with remission of AML. C1 and C2 show that the thickening of choroid was reversed after remission of AML.

**Figure 4:**
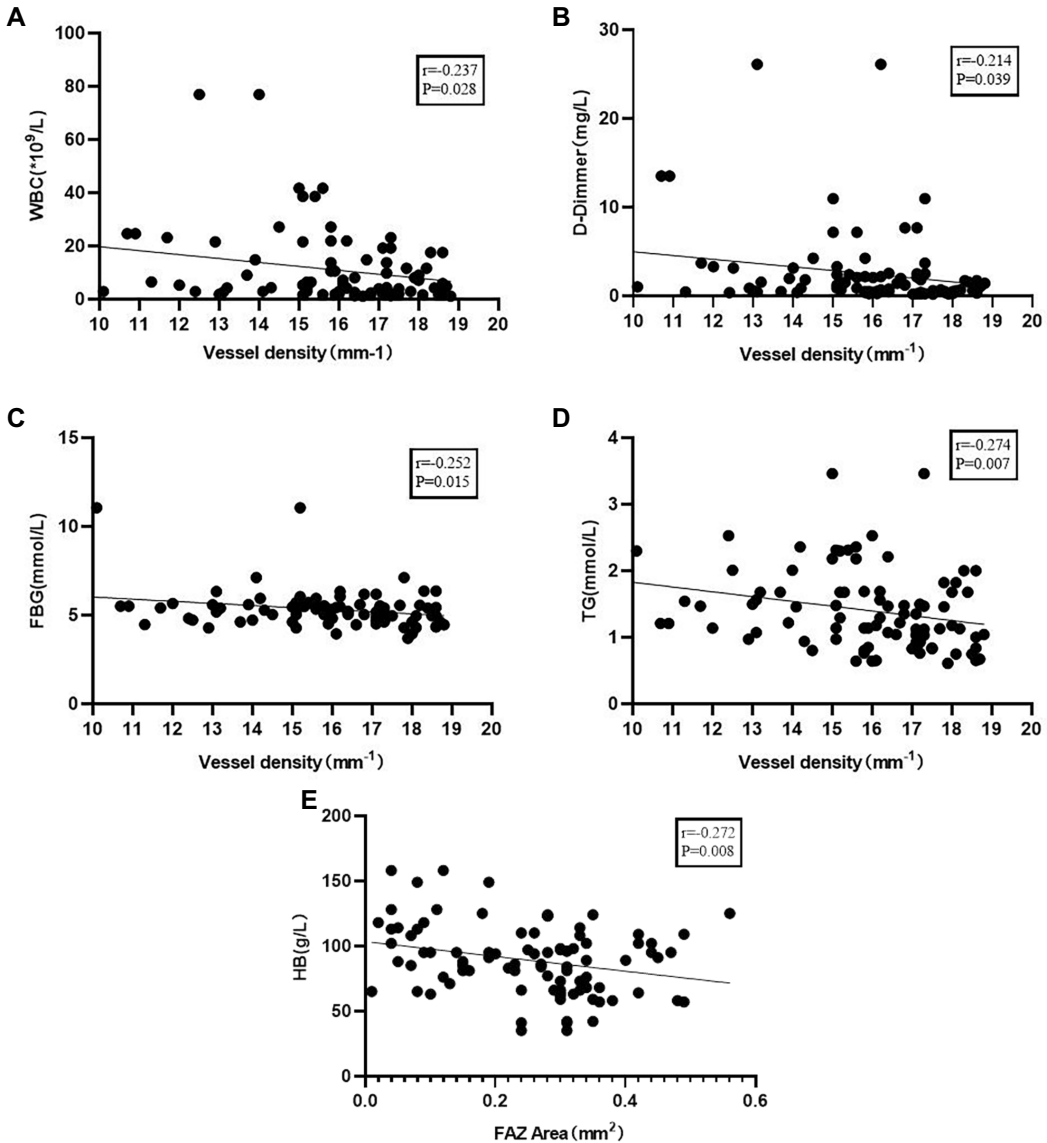
**(A)** Correlation of vessel density with white blood cells (WBC). **(B)** Correlation of vessel density with D-dimmer. **(C)** Correlation of vessel density with fasting plasma glucose (FBG). **(D)** Correlation of vessel density with triglyceride (TG). **(E)** Correlation of foveal avascular zone (FAZ) area with HB.

To determine the potential influencing factors associated with the above parameters, Spearman’s correlation coefficient was calculated using the clinical variables from all 93 eyes. VD in the macular region was lower in patients with higher WBC (r = −0.217, *p* = 0.036), D-dimer (*r* = −0.279, *p* = 0.001), FBG (*r* = −0.298, *p* = 0.004), and TG (*r* = −0.336, *p* = 0.001) levels. FAZ area was negatively correlated with HB (*r* = −0.258, *p* = 0.012; Figure 4).

## Discussion

Most ocular manifestations in patients with leukemia are not due to direct infiltration of the disease but rather changes in hematologic parameters that may lead to hemorrhage (11). Our study showed that among patients with AML, those who developed leukemic retinopathy had lower HB counts, higher WBC counts, and higher percentages of circulating blasts. Nevertheless, PLT counts did not appear to be related. In previous research, some results were consistent with ours, but some differed (12). El-asrar et al. found that patients with AML with Roth plaques had significantly higher WBC counts than did those without hemorrhage and that thrombocytopaenia was not associated with retinal hemorrhage (13). Some researchers have suggested that low HB in patients with AML is associated with intraretinal hemorrhage (3, 14). According to Guyer et al. patients with acute leukemia and retinopathy have higher levels of anemia and percentages of circulating blasts and lower PLT counts than do those without retinopathy (15). Contrary to our findings, Wechsler et al. and Zhuang et al. found a statistically significant association between retinal hemorrhage and thrombocytopaenia (16, 17). A recent study of acute leukemia showed that patients with clinically visible leukemic retinopathy had higher WBC counts and fewer PLTs than did those without retinal signs (18). The sample size and classification of leukemia may be a result of differences in findings. A high proportion of patients in our study had abnormally high FDP and D-dimer levels. The onset of leukemia was associated with hemostatic derangement, favoring hypercoagulability. Coagulopathy was due to thrombin activation. This is evidenced by the increased D-dimer level (19). A higher D-dimer level may be a risk factor for leukemic retinopathy. Further research is required to obtain more conclusive data.

Optical coherence tomography can identify the blood flow information of the retina and choroid with high resolution, image retinal and choroidal microvascular circulation in living tissue (20, 21), require no contrast agent, and avoid allergies and various contraindications. OCTA may have unique advantages in the management and follow-up of patients with leukemia with its non-invasive and quantitative analysis function.

This study evaluated changes in superficial retinal perfusion in patients with AML. We found that retinal perfusion in the macular region was significantly reduced in the acute phase of the disease, except for the central subfield, in patients with AML than in individuals with healthy eyes, which is consistent with the findings of Cicinelli et al. (18); they also found lower VD in the SCP adjacent to the fovea in patients with acute leukemia, with no significant change in the fovea. This change was less pronounced in the central subfield, possibly because there are fewer vessels. Acute leukemic anemia, thrombocytopaenia, and a high-viscosity state due to an increased WBC count result in an abnormal expansion of retinal capillaries and loss of physiological branching patterns. The velocity of blood in the vasculature has an inverse relationship with the vessel cross-sectional area (volumetric flow rate = flow velocity × cross-sectional area). We hypothesized that a decrease in blood flow velocity below the minimum OCTA detection threshold might cause loss of blood flow signals (22).

Comparing fundus photographs before and after treatment, we found that most patients had no significant changes, but their retinal perfusion had begun to improve. Previous studies have also suggested that retinal changes secondary to leukemic retinopathy are mostly transient and will subside without permanent damage. Abnormal retinal venous blood flow velocity was observed in patients with chronic myeloproliferative neoplasms which returned to normal levels after cytoreductive therapy (23). After leukemia remission, the perfusion of the capillaries in the macula and peripapillary areas improved (18), suggesting that the damage might be reversible. These studies revealed subclinical changes in the eyes of patients with acute leukemia, which can reflect the progression of systemic disease and may be helpful for the evaluation of patient efficacy and follow-up.

In addition, VD decreased significantly in patients with higher WBC counts, and the FAZ area was larger in patients with lower HB. This indicates that patients with more severe bone marrow damage have a greater reduction in retinal perfusion. VD decreased as D-dimer levels increased. Elevated D-dimer levels are common in acute leukemia, and their relationship with retinal circulation requires further clarification (24, 25). Elevated blood glucose and TG levels can also lead to a decrease in VD. VD is known to decrease in patients with diabetes (26). A drop in blood flow caused by hyperlipidemia has also been confirmed. This suggests that maintaining normal blood glucose and lipid levels also benefits patients with AML.

Histologic examination revealed choroidal leukemic infiltrates in the eyes of up to 65% of patients with leukemia and 31% of patients with fatal leukemia (27). This makes it the most common site of ocular involvement in leukemia (28). Previous studies have shown that the choroid in patients with acute leukemia thickened owing to leukocyte infiltration and became thinner after chemotherapy (29, 30). None of the patients with AML in our study had significant clinical signs in the macular region, but an increase in ChT was observed. In the acute phase of leukemia, choroidal invasion results in reduced capillary blood flow and increased ChT (31, 32). The causes of choroidal thickening can be explained as follows: leukemic cells adhere to the inner wall of choroidal vessels; extravasated leukemic cells compress the vessels, resulting in a reduction in choroidal blood flow velocity; and an increased inflow of fluids into the choroidal interstitial tissue due to blood flow congestion and choroidal infiltration of leukemia cells occurs, causing choroidal thickening. During the active phase of the disease, leukemic cells may lie dormant in the choroid and then subside as the treatment progresses. In patients with Vogt-Koyanagi-Harada syndrome, the choroid thickens almost a month before an anterior uveal recurrence in the context of recurrence, even before the appearance of any fundus signs of posterior involvement (33). A longer follow-up is needed to determine whether choroidal changes in patients with acute leukemia have similar clinical values.

In conclusion, patients with AML appeared to have subclinical retinal perfusion loss and choroidal thickening in the acute phase of the disease, and they partially recovered with remission. Injury to the bone marrow function may cause a decrease in retinal perfusion. Leukemic retinopathy is associated with abnormal hematologic parameters and coagulopathy. Because of the short follow-up period in this study, these changes could not be traced when the patients relapsed. The utility of OCTA in revealing subclinical ocular involvement and monitoring treatment response and the risk of relapse in patients with acute leukemia requires further evidence. Owing to the limitations of the OCTA software version, only superficial VD was analyzed, which was a disadvantage of this study.

## Data availability statement

The raw data supporting the conclusions of this article will be made available by the authors, without undue reservation.

## Ethics statement

The studies involving human participants were reviewed and approved by the Ethics Committee of the Ruijin Hospital Luwan Branch. The patients/participants provided their written informed consent to participate in this study. Written informed consent was obtained from the individual(s) for the publication of any potentially identifiable images or data included in this article.

## Author contributions

LY designed the study, analyzed the data, and wrote the manuscript. YC, TS, and YZ recruited the patients and collected the data. XS conceived the study and performed a critical revision of the manuscript for intellectual content. All authors contributed to the article and approved the submitted version.

## Funding

This project was supported by the Research Foundation of Huangpu District, Health Commission, Shanghai, China (HLM202008).

## Conflict of interest

The authors declare that the research was conducted in the absence of any commercial or financial relationships that could be construed as a potential conflict of interest.

## Publisher’s note

All claims expressed in this article are solely those of the authors and do not necessarily represent those of their affiliated organizations, or those of the publisher, the editors and the reviewers. Any product that may be evaluated in this article, or claim that may be made by its manufacturer, is not guaranteed or endorsed by the publisher.
